# Incorporating causal factors into reinforcement learning for dynamic treatment regimes in HIV

**DOI:** 10.1186/s12911-019-0755-6

**Published:** 2019-04-09

**Authors:** Chao Yu, Yinzhao Dong, Jiming Liu, Guoqi Ren

**Affiliations:** 1 School of Computer Science and Technology, Dalian University of Technology, No. 2, Linggong Road, Dalian, 116024 China; 2 Department of Computer Science, Hong Kong Baptist University, Kowloon Tong, Hong Kong, China

**Keywords:** Reinforcement learning, Dynamic treatment regime, HIV, Causal factors

## Abstract

**Background:**

Reinforcement learning (RL) provides a promising technique to solve complex sequential decision making problems in health care domains. However, existing studies simply apply naive RL algorithms in discovering optimal treatment strategies for a targeted problem. This kind of direct applications ignores the abundant causal relationships between treatment options and the associated outcomes that are inherent in medical domains.

**Methods:**

This paper investigates how to integrate causal factors into an RL process in order to facilitate the final learning performance and increase explanations of learned strategies. A causal policy gradient algorithm is proposed and evaluated in dynamic treatment regimes (DTRs) for HIV based on a simulated computational model.

**Results:**

Simulations prove the effectiveness of the proposed algorithm for designing more efficient treatment protocols in HIV, and different definitions of the causal factors could have significant influence on the final learning performance, indicating the necessity of human prior knowledge on defining a suitable causal relationships for a given problem.

**Conclusions:**

More efficient and robust DTRs for HIV can be derived through incorporation of causal factors between options of anti-HIV drugs and the associated treatment outcomes.

## Background

*Reinforcement learning* (RL) [1] has achieved tremendous achievements in solving complex sequential decision making problems in various health care domains, such as treatment in HIV [2], cancer [3], diabetics [4], schizophrenia [5], and sepsis [6]. In such typical RL implementations, a model designer normally formulates the learning components (the objective, state, action and reward etc.), specifies the presentation and efficiency techniques, and then simply lets the RL algorithms run until a satisfactory solution is obtained. Such fully automated and black-box learning processes ignore rich knowledge encoded in causal relationships between variables like duration, dose or type of treatments, and the corresponding therapeutic outcomes. Thus, the learned policies may not be interpretable enough to explain why some policies are helpful while others are not [7].

Discovering effective treatment strategies for HIV-infected individuals remains one of the most significant challenges in medical research. To date, the effective way to treat HIV makes use of a combination of anti-HIV drugs (i.e., antiretrovirals) in the form of *Highly Active Antiretroviral Therapy* (HAART) to inhibit the development of drug-resistant HIV strains [8]. Patients suffering from HIV are typically prescribed a series of treatments over time in order to maximize the long-term positive outcomes of reducing patients’ treatment burden and improving adherence to medication. However, due to the differences between individuals in their immune responses to treatment at a particular time, discovering the optimal drug combinations and scheduling strategy is a difficult task in both medical research and clinical trials. In this paper, we propose a *causal policy gradient* (CPG) algorithm that is able of incorporating causal factors into an RL process in order to facilitate the final learning performance and increase explanations of learned strategies. We illustrate how CPG can be applied to solve DTRs problems in HIV. Experiments prove the effectiveness of CPG in designing more efficient and robust treatment protocols in HIV. The remaining paper is organized as follows. We first discuss some related work and introduce the main principle of CPG algorithm. We then provide the details of implementation of CPG in HIV treatment. Finally, we conclude the paper by pointing out some directions for future work.

## Related work

RL has been applied to DTRs in HIV by several studies. Ernst et al. [9] first introduced RL techniques in computing *Structured Treatment Interruption* (STI) strategies for HIV infected patients. Using a mathematical model [8] to artificially generate the clinical data, a batch RL method, i.e., *fitted Q iteration* (FQI) with extremely randomized trees, was applied to learn an optimal drug prescription strategy in an off-line manner. The derived STI strategy is featured with a cycling between the two main anti-HIV drugs: *Reverse Transcriptase Inhibitors* (RTI) and *Protease Inhibitors* (PI). Using the same mathematical model, Parbhoo [10] further implemented three kinds of batch RL methods, FQI with extremely randomized trees, neural FQI and *least square policy iterations* (LSPI), to the problem of drug scheduling and HIV treatment design. Results indicated that each learning technique had its own advantages and disadvantages. Moreover, a testing based on a ten-year period of real clinical data from 250 HIV-infected patients in Charlotte Maxeke Johannesburg Academic Hospital, South Africa, verified that the RL methods were capable of suggesting treatments that are reasonably compliant with those suggested by clinicians.

The authors in [11] used the Q-learning algorithm in HIV treatment and obtained a good performance and high functionality in controlling the free virions for both certain and uncertain HIV models. A mixture-of-experts approach was proposed in [2] to combine the strengths of both kernel-based regression methods (i.e., history-alignment model) and RL (i.e., model-based Bayesian POMDP model) for HIV therapy selection. Making use of a subset of the EuResist database consisting of HIV genotype and treatment response data for 32,960 patients, together with the 312 most common drug combinations in the cohort, the treatment therapy derived by the mixture-of-experts approach outperform those derived by using each method alone. Marivate et al. [12] formalized a routine to accommodate multiple sources of uncertainty in batch RL methods to better evaluate the effectiveness of treatments across subpopulations of HIV patients. Killian et al. [13] similarly attempt to address and identify the variations across subpopulations in the development of HIV treatment policies by transferring knowledge between task instances.

Unlike the above studies that mainly focus on value-based RL for developing treatment policies in HIV, we are the first to evaluate policy gradient RL methods in such problems. Moreover, in this paper, we aim at modeling causal relationships between the options of anti-HIV drugs and the associated treatment effect, and introducing such causal factors into policy gradient learning process, in order to facilitate the final learning process and increase its interpretation.

## Methods

In this section, we first provide basic introduction to RL and particularly the policy gradient RL, and then present the main procedure of the proposed causal policy gradient algorithm.

### Policy gradient RL

RL enables an agent to learn effective strategies in sequential decision making problems by trial-and-error interactions with its environment [1]. The *Markov decision process (MDP)* has been used to formalize an RL problem, which has a long history in the research of *theoretic decision making* in stochastic settings. Formally, an MDP can be defined by a 5-tuple $\mathcal {M} = \left (\mathcal {S}, \mathcal {A}, \mathcal {P}, \mathcal {R},\gamma \right)$, where $\mathcal {S}$ is a finite *state* space, and $s_{t}\in \mathcal {S}$ denotes the state of the agent at time *t*; $\mathcal {A}$ is a set of *actions* available to the agent, and $a_{t}\in \mathcal {A}$ denotes the action that the agent performs at time *t*; $\mathcal {P}(s,a,s^{\prime }): \mathcal {S} \times \mathcal {A} \times \mathcal {S} \rightarrow [0,1]$ is a Markovian *transition function* when the agent transits from state *s* to state *s*^′^ after taking action *a*; $\mathcal {R}: \mathcal {S} \times \mathcal {A} \rightarrow \mathfrak {R}$ is a *reward function* that returns the immediate reward $\mathcal {R}(s,a)$ to the agent after taking action *a* in state *s*; and *γ*∈ [ 0,1] is a *discount factor*.

An agent’s *policy*$\pi : \mathcal {S} \times \mathcal {A} \rightarrow [0,1]$ is a probability distribution that maps an action $a \in \mathcal {A}$ to a state $s \in \mathcal {S}$. When given an MDP and a policy *π*, the expected reward of following this policy when starting in state *s*, *V*^*π*^(*s*), can be defined as follows:

1 $$\begin{array}{@{}rcl@{}} V^{\pi}(s) = E_{\pi} \left[\sum_{t=0}^{\infty}\gamma^{t}\mathcal{R}(s_{t},\pi(s_{t}))|s_{0}=s\right] \end{array} $$

and can also be defined recursively using the *Bellman operator*$\mathscr {B}^{\pi }$:

2 $$\begin{array}{@{}rcl@{}} \mathscr{B^{\pi}}V^{\pi} = \mathcal{R}(s,\pi(s)) + \gamma \sum_{s^{\prime}\in \mathcal{S}}\mathcal{P}(s,a,s^{\prime})V^{\pi}(s^{\prime}). \end{array} $$

Since the *Bellman operator*$\mathscr {B}^{\pi }$ is a contraction mapping of value function *V*, there exists a fixed point of value *V*^*π*^ such that $\mathscr {B}^{\pi }V^{\pi }=V^{\pi }$ in the limit. The goal of an MDP problem is to compute an *optimal policy* *π*^∗^ such that $V^{\pi ^{\ast }}(s)\geq V^{\pi }(s)$ for every policy *π* and every state *s*∈*S*.

Broadly, there are mainly two types of solutions to an RL problem: the value-function based solutions that maintain a value function whereby a policy can be derived, and the direct policy search solutions that try to estimate the policy directly without representing a value function explicitly [14]. The former include the model-based dynamic programming methods such as *value iterations* (VI) and *policy interactions* (PI), or direct RL methods such as *temporal difference* (TD) methods (e.g., Q-learning [15]). Direct *policy gradient* (PG) is typical policy search method, which can parameterize the policy and estimate the gradient relative to policy parameters. Its update rule is given as follows:

3 $$\begin{array}{@{}rcl@{}} \bigtriangledown_{\theta}U (\theta)\leftarrow \frac{1}{m}\sum_{i=1}^{m}\bigtriangledown_{\theta}log\pi_{\theta}(\tau,\theta)R(\tau) \end{array} $$

where *τ* is the trajectory, *θ* is the parameter, and *m* is the number of trajectories.

In Eq. (3), ▽_*θ*_*U*(*θ*) is the gradient of the policy, *π*_*θ*_(*τ*,*θ*) is the probability of the occurrence of a trajectory(*τ*), ▽_*θ*_*logπ*_*θ*_(*τ*,*θ*) is the steepest direction when *τ* changes with *θ*, and *R*(*τ*) is the reward of a trajectory to control the updating direction and step size of parameter.

### Incorporating causal factors into policy gradient RL

The direct PG algorithm only considers each state and expected value of the actions, but does not relate any causal effect between the actions and final performance. This is contradictory to the fact that, in many fields, there exhibit various kinds of correlations between actions and corresponding outcomes. This is more prominent in the medical area where different options such as medicine dosage, treatment type and duration would usually give rise to various treatment outcomes of patients. To model this kind of causal relationships, we introduce a causal factor *C*_(*B*|*A*)_ of event B due to event A as follows:

4 $$\begin{array}{@{}rcl@{}} C_{(B|A)}=P(B/A)-P(B/^{\neg} A) \end{array} $$

where *P*(*B*/*A*) is the probability of event B conditioned on event A, and *P*(*B*/^¬^*A*) is the probability of event B given that event A did not happen. Expanding Eq. (4) gets the following equation:

5 $$\begin{array}{@{}rcl@{}} C_{(B|A)}=P(A \cap B)/P(A)-P(^{\neg} A \cap B)/(1-P(A)) \end{array} $$

where *P*(*A*) represents the probability of occurrence of event A, *P*(*A*∩*B*) represents the probability of event A and event B occurring at the same time, and *P*(^¬^*A*∩*B*) represents the probability that event A does not happen, but event B happens at the same time. The causal factor *C*_(*B*|*A*)_ can be computed using a sampling method proposed in [16].

If causal factor *C* is positive, there is a causal relationship between event *A* and event *B*, because event *B* occurred because of *A* (that is, event *A* is the cause of event *B*) and negative otherwise. Causal factors *C* can be incorporated into the policy gradient learning process as follows:

6 $$\begin{array}{@{}rcl@{}} \bigtriangledown_{\theta}U (\theta)\leftarrow \frac{1}{m}\sum_{i=1}^{m}\bigtriangledown_{\theta}log\pi_{\theta}(\tau,\theta)*R(\tau)*C \end{array} $$

where *τ*, *θ*, ▽_*θ*_*U*(*θ*), *π*_*θ*_(*τ*,*θ*), and ▽_*θ*_*logπ*_*θ*_(*τ*,*θ*) are the same as Eq. (3).

The product of *C* and *R*(*τ*) controls the updating direction and step size of parameters, in order to indicate how causes (i.e., decisions along the trajectory) affect the final performance for each trajectory. Table 1 gives the full sketch of the proposed CPG algorithm based on the Monte Carlo policy gradient method ERINFORCE [17] that has decomposed the trajectory into states and actions.

**Table 1:** The Causal Policy Gradient (CPG) Algorithm

Algorithm 1: The CPG Algorithm
Function CPG
Input: a differentiable policy parameterizations *π*(*a*|*s*,*θ*), ∀*a*∈A, s ∈S, *θ*∈*R*^*d*^, C=0;
Initialize policy parameter *θ*;
Repeat forever:
Define event A and event B;
Generate an episode *s*_0_,*a*_0_,*r*_1_,...,*s*_*T*−1_,*a*_*T*−1_,*r*_*T*_, following *π*(*a*|*s*,*θ*);
For each step of the episode t=0,...,T-1:
G ← average future return from step t;
*C*=*P*(*A*∩*B*)/*P*(*A*)−*P*(^¬^*A*∩*B*)/*P*(1−*P*(*A*));
*θ*←*θ*+*α*▽_*θ*_*logπ*(*a*_*t*_|*s*_*t*_,*θ*)∗*G*∗*C*;
End for
Return *θ*;
End CPG

## Results

In this section, we evaluate CPG in the treatment of HIV to verify its effectiveness. We first briefly introduce the DTR problem in HIV and its RL formulations. We then use the direct PG algorithm to simulate HIV treatment, and investigate how the proposed CPG algorithm can be applied to solve this problem. Finally, we provide some discussions on the shortcomings of current research that need to be addressed in the future work.

### MDP for DTRs in HIV

The simulated HIV treatment model [9] consists of a six dimensional continuous state space, including the concentrations of healthy *CD*^4+^ T-lymphocytes (*T*_1_), healthy macrophages (*T*_2_), healthy infected *CD*^4+^ T-lymphocytes $\left (T^{*}_{1}\right)$, infected macrophages ($T^{*}_{2}$), free virus particles (*V*) and HIV-specific cytotoxic T-cells (*E*). The full drug interaction model is given by the Appendix.

While anti-retroviral treatment regimens are sometimes augmented by other types of drugs that enhance the effect of anti-HIV treatment, bolster the immune system, or reduce side effects, our current effort focuses on representatives of two main classes of enzymes: *reverse transcriptase inhibitor* (RTI) and *protease inhibitor* (PI). RTI prevents HIV RNA from being converted into DNA, thus blocking integration of the viral code into the target cell. On the other hand, PI affects the viral assembly process in the final stage of the viral life cycle, preventing the proper cutting and structuring of the viral proteins before their release from the host cell. PI therefore effectively reduces the number of infectious virus particles released by an infected cell. In all, there are four treatment regimens: only RTI on, only PI on, RTI and PI on, RTI and PI off. The four medication regimens are treated as four discrete actions.

The reward of the process at time t can be defined as $1000E_{t}-0.1V_{t}-20000\xi _{1t}^{2}-2000\xi _{2t}^{2}$ [9]. The *ξ*_1*t*_ is set to 0.7 when the RTI is cycled on, while *ξ*_2*t*_ is set to 0.3 when PI is cycled on; and the *ξ*_1*t*_ or *ξ*_2*t*_ is set to 0, when RTI or PI is off. The formula of the reward value indicates that the increase of *E* or the decrease of *V* is conducive to obtaining larger rewards (i.e., promoting the treatment of HIV), while excessive application of enzymes would damage the cells and thus decrease the reward. As shown in [9], in the absence of treatment, the model has three equilibrium points as given in Table 2.

**Table 2:** Different equilibrium points of the six cells

Equilibrium point	T_1_	T_2_	T$^{*}_{1}$	T$^{*}_{2}$	V	E
The healthy, unstable state	10^6^	3198	0	0	0	10
The healthy, locally stable state	967839	621	76	6	415	353108
The non-healthy, locally stable state	163573	5	11945	46	63919	24

### RL for HIV treatment

**Fig. 1:**
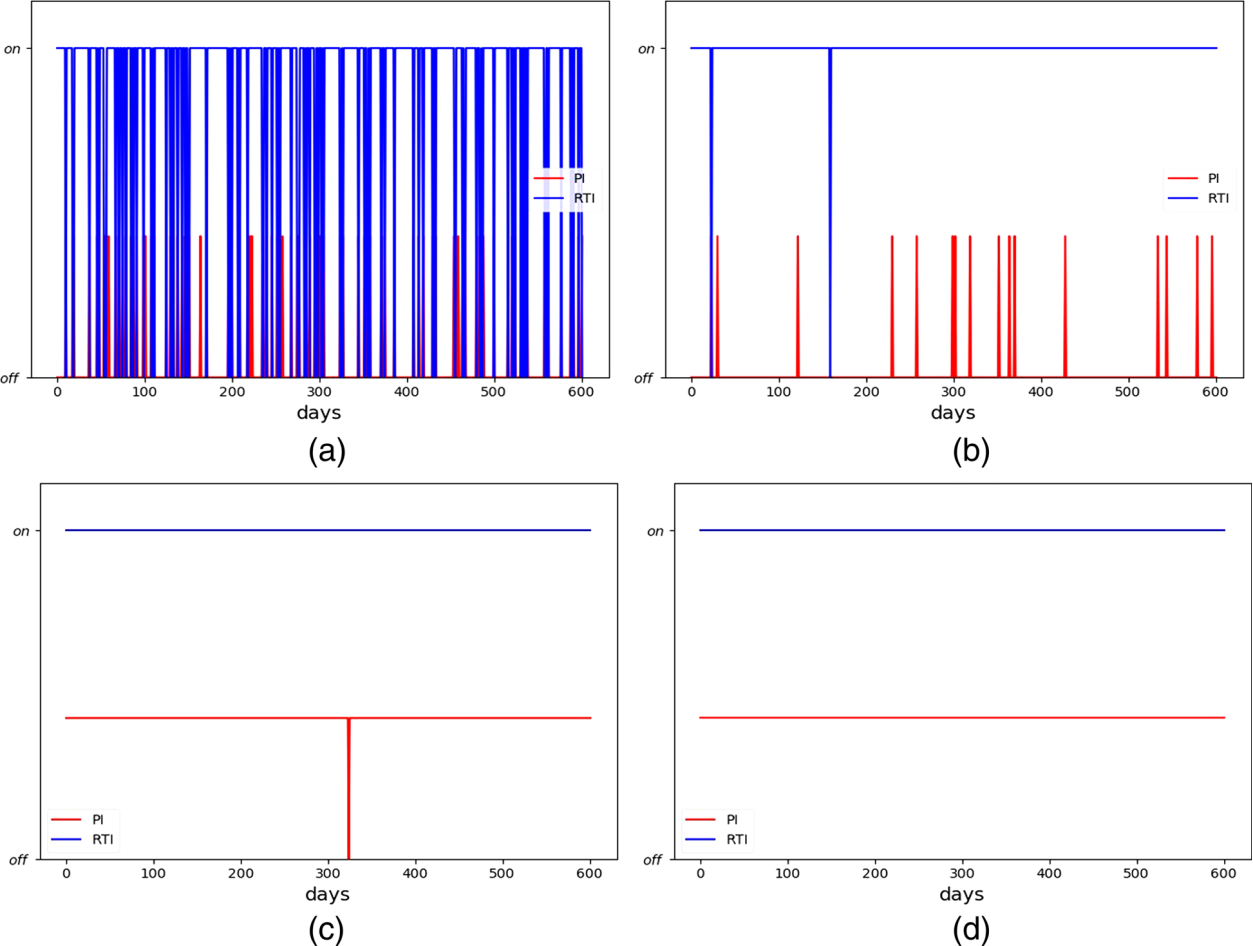
The medication regimen **a** before learning; **b**-**c** during learning; and **d** after learning

We first apply the direct PG to the treatment of HIV. The non-healthy locally stable equilibrium point was taken as the initial state. During the experiment, the drug is taken every 5 days and a course of treatment is observed for 600 days. Supposing there are 300 patients, each patient is in the unhealthy state initially. The decay factor *γ* is 0.85, and the learning rate *λ* is 0.02. For the first patient, we used the policy gradient algorithm to generate a random strategy. Figure 1 shows the random DTR for the first patient and the continuously learned DTR for other patients during the Simulated HIV Treatment. For the first patient (Fig. 1a), RTI and PI were randomly chosen, indicating an irregular therapeutic process. The RL algorithm gradually learned that continuous use of RTI would have a significant healing effect on HIV (Fig. 1b-c). For the 300th patient (Fig. 1d), the algorithm finally learned a strategy that the RTI and PI were continuously used throughout the treatment.

**Fig. 2:**
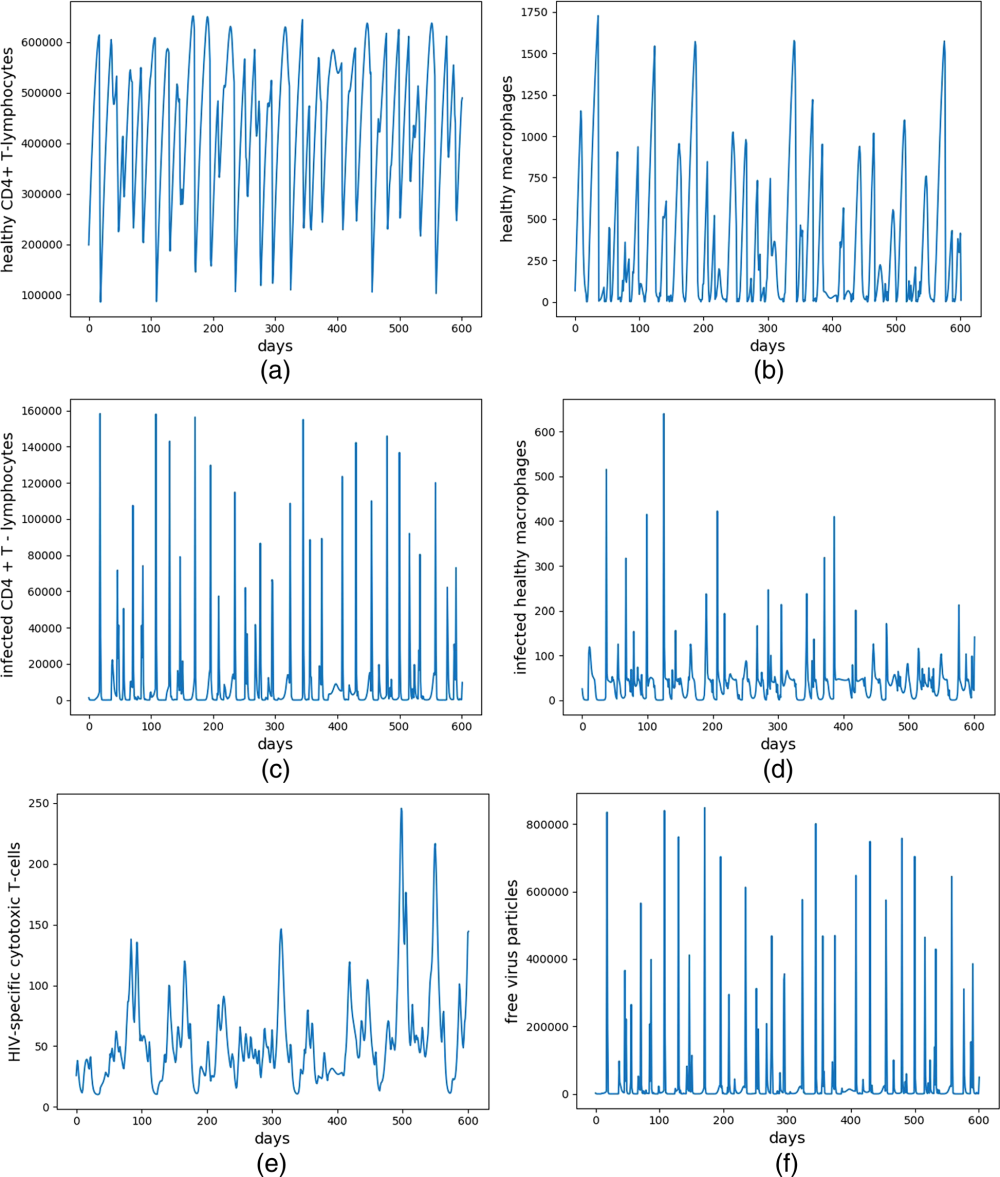
The evolution of the six types of cells for the first patient (i.e., before learning). **a**-**f** corresponds to the continuous change of $\mathrm {T}_{1}, \mathrm {T}_{2}, \mathrm {T}_{1}^{*}, \mathrm {T}_{2}^{*}$, E and V cells, respectively

Figure 2 shows the continuous change in the number of six cells (*T*_1_, *T*_2_, $T^{*}_{1}$, $T^{*}_{2}$, *V*, *E*) over time for the first patient (i.e., before learning). The number of each cell in Fig. 2a-f fluctuate greatly, and the number of cells change irregularly. The number of *T*_1_, *T*_2_ and *E* do not increase significantly, and the number of $T^{*}_{1}$, $T^{*}_{2}$ and *V* also did not decrease much. Therefore, this treatment effect is very poor because the patient’s condition has not improved. The patient was still in a non-healthy state after one course of treatment.

**Fig. 3:**
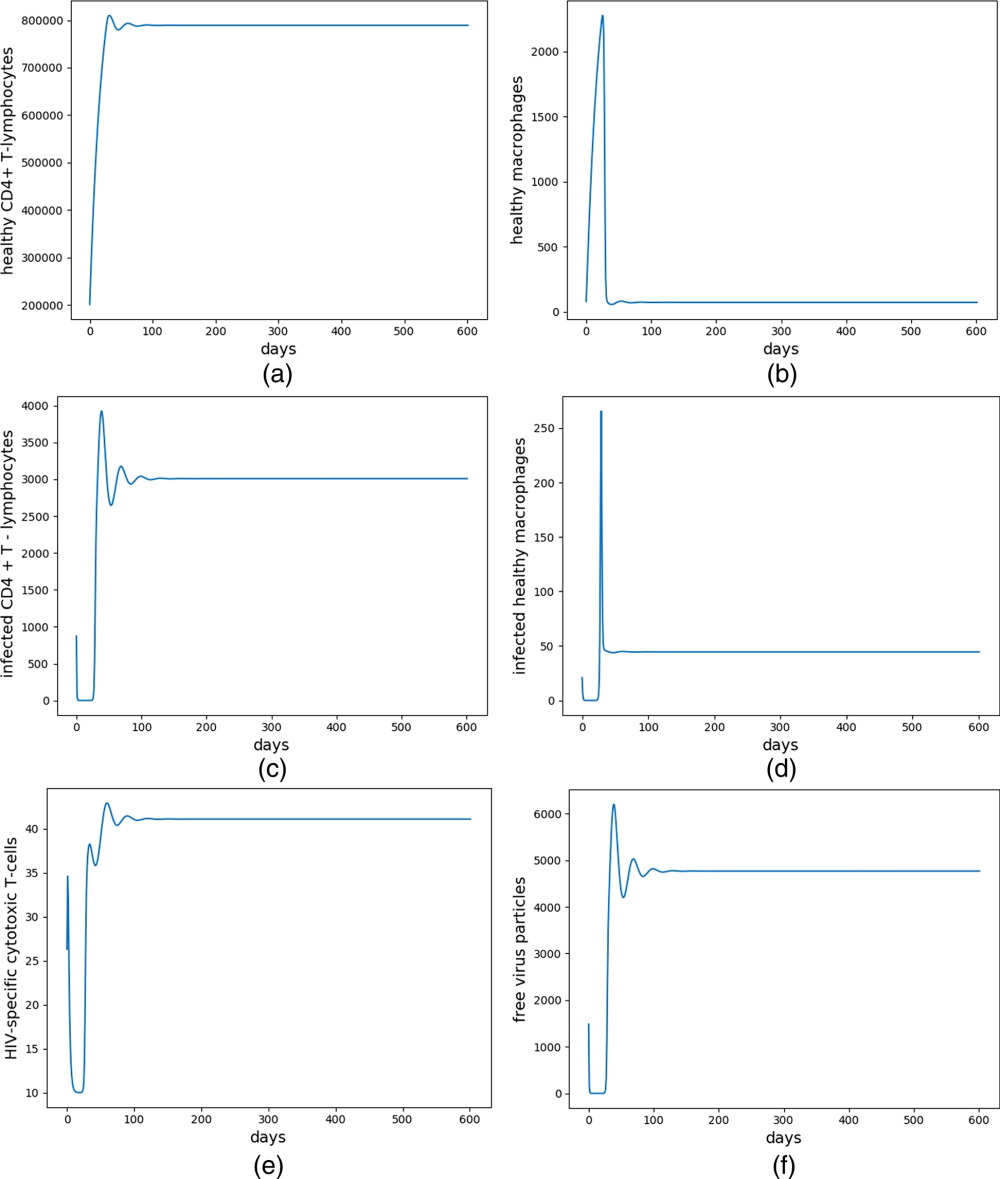
The evolution of the six types of cells after learning over 300 patients. **a**-**f** corresponds to the continuous change of $\mathrm {T}_{1}, \mathrm {T}_{2}, \mathrm {T}_{1}^{*}, \mathrm {T}_{2}^{*}$, E and V cells, respectively

Figure 3 shows the continuous change in the number of six cells after learning over 300 patients. As shown in Fig. 1d, the patient has been administered by two enzymes (RTI and PI) continuously after learning and the number of each cell in Fig. 3 has regular change. After 100 days of treatment, *T*_1_, *T*_2_ and *E* increase significantly compared to the initial state, while the number of $T^{*}_{1}$, $T^{*}_{2}$ and *V* cells reduce significantly. In the end, the number of the six cells reach to a dynamic equilibrium as follows: (*T*_1_, *T*_2_, $T^{*}_{1}$, $T^{*}_{2}$, *V*, E) ≈ (800000, 100, 3000, 50, 5000, 40). Therefore, this treatment has a better therapeutic effect because the patient’s health condition has improved. However, compared with the non-healthy locally stable equilibrium point, the number of *T*_1_, *T*_2_ and *E* cells were still low, while the number of the other three kinds of cells were still highly harmful to humans. The patients were still in a transitional state of non-health to healthy state after the treatment. At this time, the patients need to keep on taking medication, because they relied on the drug to maintain the current healthy state. Once the medication was stopped, the number of harmful *V* cells may rebound greatly.

**Fig. 4:**
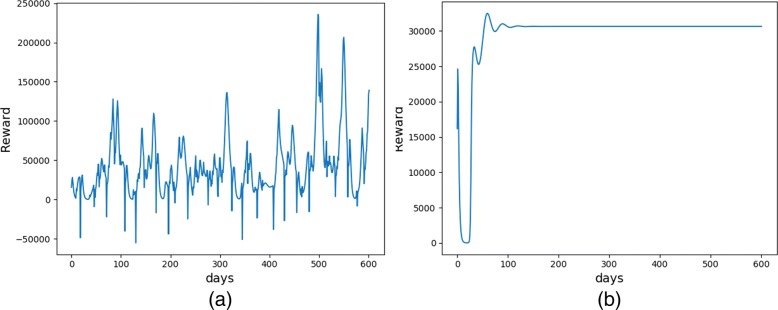
The evolution of reward of **a** the first patient; and **b** the 300th patient

Figure 4 shows the patients’ reward at each decision step before learning and after learning. The reward before learning fluctuates greatly, even emerging negative value, which indicates the ineffectiveness of initial treatment strategy. The reward after learning converges to a higher value, and finally stabilized at around 3800. Thus, the medication regimen has a better therapeutic effect after using the policy gradient RL method.

### Applying CPG for HIV treatment

Let the initial state, parameter value, observation period, patient number, initial strategy and other relevant variables be the same as “RL for HIV treatment” section. We also apply CPG to the HIV model. To define the causal factor, let event *A* represent the action taken each time (i.e., adding enzyme RTI or PI), event ^¬^*A* mean no enzyme action, and event *B* mean the outcome of *V*>415 (i.e., the number of free virus particles is greater than 415). Thus, *P*(*A*) indicates the probability of taking an enzymatic action at each time, *P*(*A*∩*B*) represents the probability of simultaneous occurrence of free virus particles being greater than 415 and adding enzyme at the same time, and *P*(^¬^*A*∩*B*) represents the probability of simultaneous occurrence of free virus particles being greater than 415 without adding enzyme. For each cause of treatment, we can count the frequencies of each event and then use these frequencies to indicate the corresponding probabilities.

**Fig. 5:**
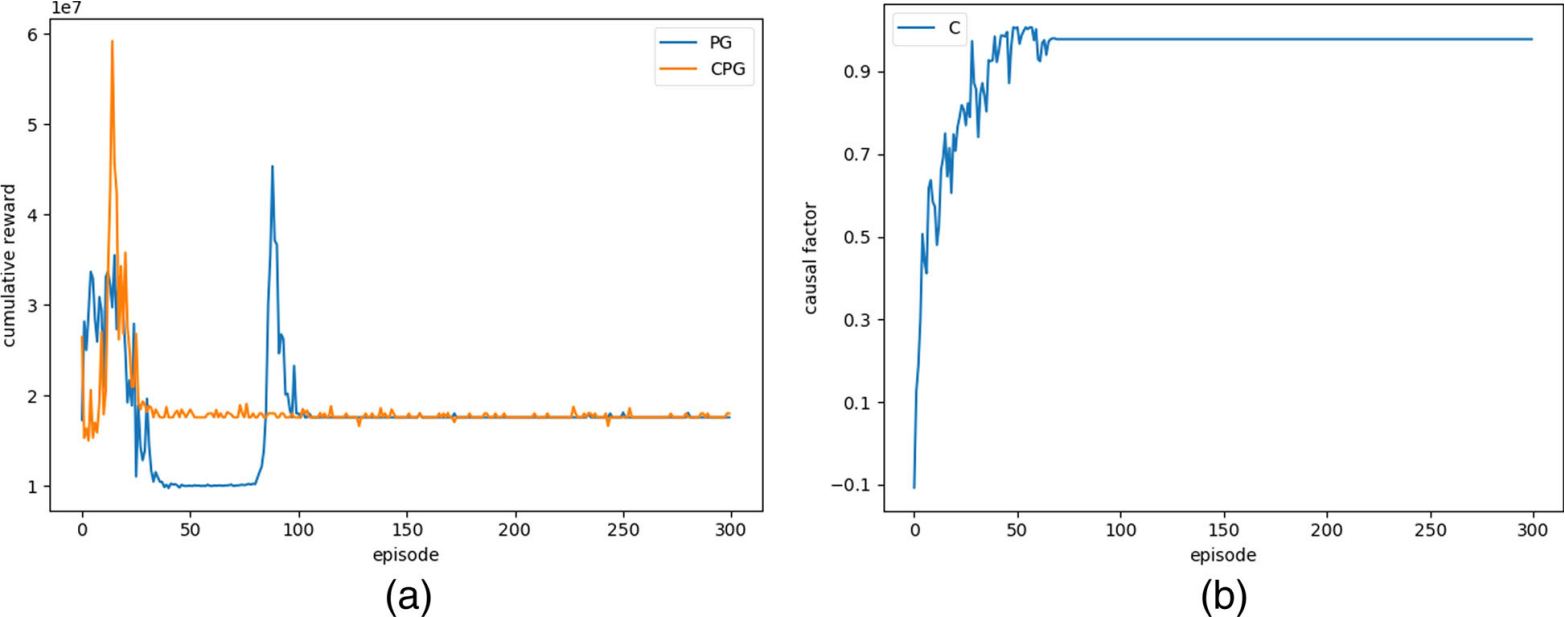
**a** Comparison of the performance of direct PG and CPG algorithm; **b** Dynamic evolution of causal factor C during learning

Figure 5a compares the performance of direct PG and CPG algorithm, where the blue and yellow line represent cumulative reward over 600 days using the direct PG algorithm and the CPG algorithm, respectively. After about 100 episodes, the final treatment strategy can be learned using the direct PG algorithm. Since the CPG algorithm can employ the causal factors to reason about the outcome of patients (i.e., how they are affected by the *V* cells) and the treatment (i.e., administration of enzyme), the policy learning process can be greatly promoted. CPG can learn the same treatment strategy as PG in less 50 episodes, indicating that the two enzymes are used continuously and the patients need to take the drug to maintain healthy. Compared to the direct PG algorithm, CPG is more efficient and robust by improving the learning speed in terms of cumulative reward and convergence rate.

Figure 5b shows the dynamic changes of causal factor (C) from the first episode to the 300th episode. During the early training stage, the causal factor is quite low, indicating little effect on the learning process. As the learning proceeds, the causal factors increase and reach a dynamic balance after around 50 episodes. Due to constantly random exploration in the learning process, the causal factor is always changing and finally close to 1. In the initial treatment phase, the patient’s *V*=63919 is much greater than 415. As learning proceeds, the medication policy became better and better and the regulative effect of causal factors also became stronger. After 50 episodes, the CPG algorithm learned the strategy of continuous dosing of both enzymes, so causal factors also reached the state of dynamic equilibrium.

The CPG algorithm requires prior definition of the causal factors in terms of causal events and outcome events. Defining different causal factors has quite different effects on the final learning performance. To test this, we defined different causal factors (*C*1 and *C*2) in Table 3.

**Table 3:** Definition of different causal factors

Event	*C*1	*C*2
A	adding enzyme RTI or PI	adding enzyme RTI or PI
^¬^A	without adding enzyme	without adding enzyme
B	*V*>415	*T*_2_<621

**Fig. 6:**
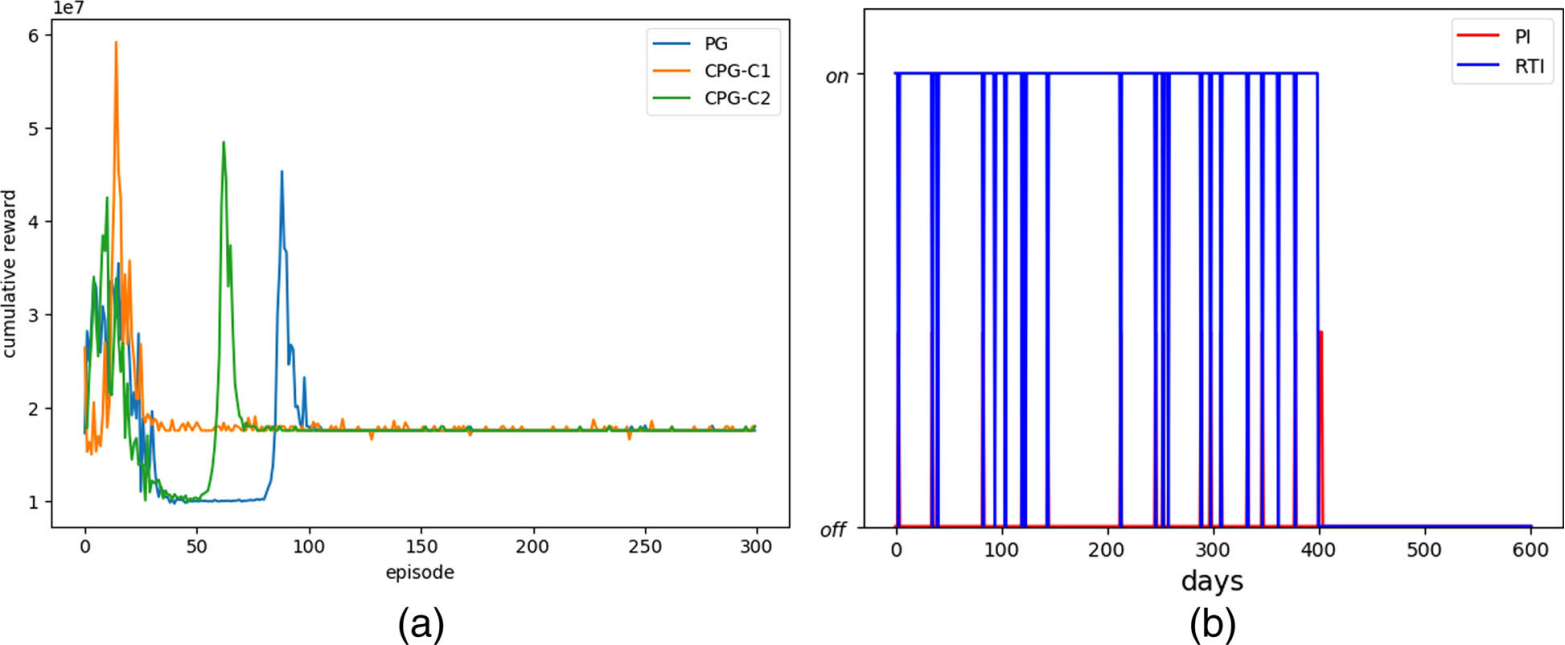
**a** Comparison of reward values for causal algorithms with different causal factors; **b** The optimum strategy in HIV treatment

Figure 6a compares the learning performance of the direct PG with different defined causal factors using CPG, in which CPG-C1 and CPG-C2 represent the CPG with causal factor C1 and C2, respectively. The main difference between C1 and C2 lies in the definition of B event. In CPG-C1, event B means that the number of free virus particles is greater than 415, while in CPG-C2, event B means that the number of healthy macrophages is less than 621. As shown in Fig. 6a, CPG-C1 has the best performance and the fastest convergence. Compared with CPG-C2, the causal factor definition of *C*1 is more reasonable (the convergence effect and convergence speed are better). In the HIV model, *V* has a greater effect on human health than *T*_2_, because *V* cells are the direct factors that influence the development of disease. Therefore, different definitions of causal factor can greatly affect the learning performance of CPG algorithm, and an appropriate causal definition can significantly speed up the performance of the algorithm.

## Discussion

Although we are able to derive an effective treatment strategy using the above direct PG and CPG algorithms, the solutions still only converged to a sub-health state that must be maintained by continuous dosing of both enzymes. In order to derive the optimal drug-free treatment strategies, we directly solve the HIV model using the Lagrangian function formula introduced in [8] (which is given by the Appendix). By solving Lagrangian function given by Eq. (17), the optimal control $\xi ^{*}_{1}$ and $\xi ^{*}_{2}$ are characterized by Eqs. (7) and (8), respectively [8]:

7 $$ {}\begin{aligned} \xi^{*}_{1}&=max(a_{1},min(b_{1},(\eta_{1}-\eta_{3}+\rho_{1}*\eta_{5})*k_{1}*V*T_{1} \\ &\quad-(\eta_{2}-\eta_{4}+\rho_{2}*\eta_{5})*f*k_{2}*V*T_{2}/2*R_{1})) \end{aligned}  $$

8 $$ {}\xi^{*}_{2}=max\left(a_{2},min\left(b_{2}, \eta_{5}*NT* \delta *\left(T^{*}_{1}+T^{*}_{2}\right)/2*R_{2}\right)\right)   $$

where *ξ*_1_∈[0, 1) and *ξ*_2_∈[0, 1) are the control variables representing RTIs and PIs, respectively. In order to get a better strategy, we set *a*_1_=0.0, *a*_2_=0.0, *b*_1_=0.7 and *b*_2_=0.3. We used partial differential equations to solve the dynamics parameters, and applied Eqs. (7) and (8) to obtain the optimal strategy. Figure 6b plots the computed optimum strategy, in which the red line and the blue line represent the dosing of PI and RTI, respectively. It is clear to see that after 400 days of treatment, the two drugs are stopped, indicating a drug-free healthy stable state of patients.

The reasons why the general RL algorithms such as PG and CPG in this paper could not discover the optimal drug-free solutions lie in two main perspectives. On one hand, the MDP model adopted in this paper only considers four discrete actions that involve two types of enzyme and assigns a predefined fixed value of 0.7 and 0.3 to the parameters of *ξ*_1_ and *ξ*_2_. This highly simplification makes it difficult or impossible to fully explore the whole state of the model in order to derive the optimal solution. Moreover, the reward function used in the MDP model is too abstract to reflect the complex dynamics of the treatments. On the other hand, the policy gradient algorithms themselves did not incorporate any sophisticated exploration strategies during the learning process. This is a critical problem since HIV treatment has long been recognized as a well-known testbed for evaluating advanced exploration algorithms in RL research [18, 19]. Previous studies have shown that the basin of attraction of the healthy steady-state in HIV is relatively small compared to the one of the non-healthy steady state. Thus, in the absence of drugs, perturbation of the uninfected steady state by adding as little as virus would lead to asymptotic convergence towards the non-healthy steady state.

## Conclusions

Simulation-based DTR design has a series of advantages over cytopathological treatment in that it can avoid the harm to the patients during the exploration of drug, provide a large amount of treatment experience for the disease with insufficient case in reality, reduce the cost of actual treatment and shorten the duration of treatment. In this paper, we investigated the role of RL in DTRs for simulated patients with HIV. We showed that both the direct PG and its causal extension could obtain a better medication regimen after a period of learning, but the CPG algorithm was more efficient and robust due to incorporation of causal factors between options of anti-HIV drugs and the associated treatment outcomes. We also showed that different definitions of the causal factor could have significant influence on the final learning performance, indicating the necessity of human prior knowledge on defining a suitable causal relationships for a given problem. How to discover the most beneficial or optimal causal factors from historical interaction trajectories is thus important to automate the whole learning process. This will be left for our future work for further investigation.

## Appendix

This mathematical model of the HIV is described by the following set of ordinary differential and Lagrangian equations:

9 $$  {}T^{\prime}_{1} = 10000-0.01T_{1}-(1-\xi_{1})*8.0*10^{-7}*V*T_{1}  $$

10 $$  {}T^{\prime}_{2} = 31.98-0.01T_{2}-(1-0.34\pi_{1})*1.0*10^{-4}*V*T_{2}  $$

11 $$  {}T^{*\prime}_{1} = (1-\xi_{1})*8.0*10^{-7}*V*T_{1}-0.7T^{*}_{1}-1.0*10^{-5}*E*T^{*}_{1}  $$

12 $$\begin{array}{*{20}l} {}T^{*\prime}_{2} &= (1-0.34\xi_{1})*1.0*10^{-4}*V*T_{2}-0.7T^{*}_{2}\\ &\quad-1.0*10^{-5}*E*T^{*}_{2} \end{array} $$

13 $$ {}\begin{aligned} V^{\prime} &= 70(1-\xi_{2})*(T^{*}_{1}+T^{*}_{2})-13V-\left[8.0*10^{-7}\right.\\ &\quad\left.*(1-\xi_{1})*T_{1}+1.0*10^{-4}*(1-0.34\xi_{1})*T_{2}\right]*V \end{aligned}  $$

14 $$  {}E^{\prime} \,=\, 1+\frac{0.3*\left(T^{*}_{1}+T^{*}_{2}\right)*E}{\left(T^{*}_{1}+T^{*}_{2}\right)+100}-\frac{0.25*\left(T^{*}_{1}+T^{*}_{2}\right)*E}{\left.T^{*}_{1}+T^{*}_{2}\right)+500}-0.1E  $$

15 $$  {}R=0.1*V+20000*\xi^{2}_{1}+2000*\xi^{2}_{2}-1000E  $$

16 $$ {} \begin{aligned} W & = W_{11}*(\xi_{1}-a_{1})+W_{12}(b_{1}-\xi_{1})+W_{21}(\xi_{2}-a_{2})\\ &\quad+W_{22}(b_{2}+\xi_{2}) \end{aligned}  $$

17 $$ {} \begin{aligned} L &= R + \eta_{1}*T^{\prime}_{1}+\eta_{2}*T^{\prime}_{2}+\eta_{3}*T^{*\prime}_{1}+\eta_{4} *T^{*\prime}_{2}+\eta_{5}*V^{\prime}\\ &\quad+\eta*E^{\prime}-W \end{aligned}  $$

where T_1_, T_2_, T$^{*}_{1}$, T$^{*}_{2}$, V, E are the number of six cells; *ξ*_1_∈[0, 1) and *ξ*_2_∈[0, 1) are the control variables representing RTIs and PIs, respectively; *W*_*ij*_>0 are the penalty multipliers; and *η*_*n*_ are the adjoint variables.

## References

[1] Sutton RS, Barto AG (1998). Reinforcement Learning: An Introduction.

[2] Parbhoo S, Bogojeska J, Zazzi M, Roth V, Doshi-Velez F (2017). Combining kernel and model based learning for hiv therapy selection. AMIA Summits Transl Sci Proc.

[3] Tseng H-H, Luo Y, Cui S, Chien J-T, Ten Haken RK, Naqa IE (2017). Deep reinforcement learning for automated radiation adaptation in lung cancer. Med Phys.

[4] Daskalaki E, Diem P, Mougiakakou SG (2016). Model-free machine learning in biomedicine: Feasibility study in type 1 diabetes. PloS ONE.

[5] Shortreed SM, Laber E, Lizotte DJ, Stroup TS, Pineau J, Murphy SA (2011). Informing sequential clinical decision-making through reinforcement learning: an empirical study. Machine learning.

[6] Weng W-H, Gao M, He Z, Yan S, Szolovits P. Representation and reinforcement learning for personalized glycemic control in septic patients. 2017. arXiv preprint arXiv:1712.00654.

[7] Hein D, Udluft S, Runkler TA (2018). Interpretable policies for reinforcement learning by genetic programming. Eng Appl Artif Intell.

[8] Adams BM, Banks HT, Kwon H-D, Tran HT (2004). Dynamic multidrug therapies for hiv: Optimal and sti control approaches. Math Biosci Eng.

[9] Ernst D, Stan G-B, Goncalves J, Wehenkel L (2006). Clinical data based optimal sti strategies for hiv: a reinforcement learning approach. 45th IEEE Conference on Decision and Control.

[10] Parbhoo S. A reinforcement learning design for hiv clinical trials. 2014. PhD thesis.

[11] Gholizade-Narm H, Noori A (2018). Control the population of free viruses in nonlinear uncertain hiv system using q-learning. Int J Mach Learn Cybern.

[12] Marivate VN, Chemali J, Brunskill E, Littman ML (2014). Quantifying uncertainty in batch personalized sequential decision making. AAAI Workshop: Modern Artificial Intelligence for Health Analytics..

[13] Killian T, Konidaris G, Doshi-Velez F. Transfer learning across patient variations with hidden parameter markov decision processes. 2016. arXiv preprint arXiv:1612.00475.

[14] Wiering M, Van Otterlo M (2012). Reinforcement learning. vol 12. Adapt Learn Optim.

[15] Watkins CJ, Dayan P (1992). Q-learning. Mach Learn.

[16] Merck CA, Kleinberg S (2016). Causal explanation under indeterminism: A sampling approach. AAAI..

[17] Williams RJ (1992). Simple statistical gradient-following algorithms for connectionist reinforcement learning. Mach Learn.

[18] Kawaguchi K (2016). Bounded optimal exploration in mdp. AAAI..

[19] Pazis J, Parr R (2013). Pac optimal exploration in continuous space markov decision processes. AAAI..

